# Genetic Characterization and Pathogenesis of Highly Pathogenic Avian Influenza Virus A (H5N1) Isolated in Egypt During 2021–2023

**DOI:** 10.3390/v17101370

**Published:** 2025-10-13

**Authors:** Mina Nabil Kamel, Yassmin Moatasim, Basma Emad Aboulhoda, Mokhtar Gomaa, Ahmed El Taweel, Omnia Kutkat, Mohamed El Sayes, Mohamed GabAllah, Hend AbdAllah, Refaat M. Gabre, Maha M. AlKhazindar, Ahmed Kandeil, Pamela P. McKenzie, Richard J. Webby, Mohamed Ahmed Ali, Ghazi Kayali, Rabeh El-Shesheny

**Affiliations:** 1 Center of Scientific Excellence for Influenza Viruses, National Research Centre, Giza 12622, Egypt; mina@human-link.org (M.N.K.); yasmin.moatasim@human-link.org (Y.M.); mokhtar.rizk@human-link.org (M.G.); ahmed.nageh@human-link.org (A.E.T.); omnia.abdelaziz@human-link.org (O.K.); mohameddiaaelsayes@outlook.com (M.E.S.); gaballah09@gmail.com (M.G.); ahmed.kandeil@stjude.org (A.K.); mohamedahmedali2004@yahoo.com (M.A.A.); 2 Department of Biotechnology, Faculty of Science, Cairo University, Cairo 12613, Egypt; rgabre@sci.cu.edu.eg; 3 Department of Anatomy and Embryology, Faculty of Medicine, Cairo University, Cairo 12613, Egypt; basma.emad@kasralainy.edu.eg (B.E.A.); hend.badawy@kasralainy.edu.eg (H.A.); 4 Department of Botany and Microbiology, Faculty of Science, Cairo University, Giza 12613, Egypt; malkhazi@aucegypt.edu.eg; 5 Department of Infectious Diseases, St. Jude Children’s Research Hospital, Memphis, TN 38105, USA; pamela.mckenzie@stjude.org (P.P.M.); richard.webby@stjude.org (R.J.W.); 6 Human Link DMCC, Dubai P.O. Box 48800, United Arab Emirates

**Keywords:** highly pathogenic avian influenza, H5N1 viruses, Egypt, wild birds, transmission, virus surveillance

## Abstract

Highly pathogenic avian influenza (HPAI) viruses have recently had a substantial impact on global poultry production and public health. In Egypt, clade 2.3.4.4b HPAI H5N1 viruses were first isolated from wild birds in 2021 and then became dominant in domestic poultry. In this study, we aimed to genetically characterize the H5N1 viruses isolated in Egypt during 2021–2023 and examine the pathogenicity and transmissibility of two H5N1 strains isolated from wild and domestic poultry in chickens. We collected 7588 specimens from live bird markets including poultry, wild birds, and environmental samples. Influenza A viruses were detected in 20.94% (484/2311) of tested samples, and 17 isolates were identified as H5N1 through complete genome sequencing. Phylogenetic analysis revealed that all H5N1 viruses were closely related to Eurasian viruses and classified into three distinct genetic groups, suggesting multiple introductions likely linked to migratory birds. Experimental infections of chickens with two H5N1 isolates, A/Pintail/Egypt/RA19853OP/2021 and A/duck/Egypt/BA20361C/2022, showed efficient replication, systemic infection, and transmission by direct contact. These findings underscore the need for continued surveillance of H5N1 at the poultry-wild bird interface to identify circulating strains, evaluate their biological characteristics, and assess their zoonotic potential.

## 1. Introduction

The high genetic variability and rapid evolution of influenza viruses, particularly avian influenza viruses (AIVs), pose significant challenges to their prevention and control [1]. AIVs cause considerable morbidity and mortality in bird populations, with severity varying by subtype. AIVs belong to the genus Alphainfluenzavirus and family Orthomyxoviridae, with eight negative-sense single-stranded enveloped RNA segments [2], coding for at least 11 structural and non-structural/regulatory proteins [3]. Based on the antigenic characteristics of their surface glycoproteins, influenza viruses are classified into 16 hemagglutinin (H1–H16) and 9 neuraminidase (N1–N9) subtypes [4,5], with two novel influenza A virus subtypes, H17N10 and H18N11, genetically detected in bats [6]. Although all AIV subtypes were detected in aquatic birds, several subtypes, including H5, H6, H7, H9, and H10, can cross the species barrier to infect mammals.

It has been almost two decades since a highly pathogenic avian influenza (HPAI) virus subtype H5N1 was first detected in Egypt. Clade 2.2.1 H5N1 was introduced into Egyptian poultry in 2006, causing outbreaks in poultry and sporadic human infections. Its endemicity and continuous circulation in Egypt have driven further genetic and antigenic variations. Through active surveillance, we have since detected additional subclades emerging in Egypt, including 2.2.1.1, 2.2.1.2, and 2.2.1.1a [7,8].

In 2016, clade 2.3.4.4b H5N8 viruses were first reported in wild birds and poultry along Egypt’s Northern coast [9] and soon became predominant across poultry sectors including backyard flocks, commercial farms, and live bird markets (LBM) [10,11,12,13]. In 2020/2021, clade 2.3.4.4b H5N1 viruses spread across Europe, Africa, Asia, and the Americas and were predominantly circulating globally [14]. In Egypt, clade 2.3.4.4b H5N1 viruses were subsequently detected in wild birds and domestic ducks from LBMs [15] where they replaced earlier H5 strains. Furthermore, the coexistence and co-circulation of clade 2.3.4.4b HPAI H5N1 and low pathogenic avian influenza (LPAI) H9N2 viruses in Egypt have raised significant concern. In 2024, new reassortant clade 2.3.4.4b H5N2 viruses were detected in duck and environmental swabs in Egypt and were found to be genetically closely related to previously circulating viruses in Egyptian poultry [16].

Several studies demonstrated that LBMs are hotspots for the emergence, maintenance, and transmission of AIVs [17,18,19]. In Egypt, the mixing of wild and domestic birds within LBMs increases the risk of reassortment between HPAI and LPAI viruses and play a role in virus evolution. Previous studies showed that several introductions of clade 2.3.4.4b H5N8 and H5N1 viruses occurred in LBMs through wild birds [9,15]. Circulation of AIVs in LBMs increases infection risk for workers and the wider public [20,21]. Serological investigation has confirmed antibodies against HPAI H5N1 in poultry workers [22].

Infection with the novel H5N1 virus raised significant concerns about the pandemic potential of the H5 subtype. Therefore, extensive and long-term surveillance of AIVs in LBMs and wild birds is essential. In this study, we aimed to characterize the emerging clade 2.3.4.4b HPAI H5N1 genotypes detected in Egypt between 2021 and 2023 through our ongoing surveillance and assess their pathogenicity, replication, and direct transmission in infected specific pathogen-free (SPF) chickens.

## 2. Materials and Methods

### 2.1. Sample Collection

Between November 2021 and March 2023, a total of 940 oropharyngeal swabs and 940 cloacal swabs were collected from seven different LBMs in the north wetlands around the Nile Delta (2 in Port Said, 3 at Damietta, 1 at Kafr El-Shaikh, and 1 at Bahira Governorate). Those were selected for their proximity to wild nesting areas from which trapped birds are sold alongside poultry within the LBMs. At each visit, cloacal and oropharyngeal swabs were collected from five birds of each species present. We also collected environmental swab samples from surfaces, birds’ drinking water, market working surfaces, animal cages, and weighing scales. All swabs were placed into phosphate-buffered saline-glycerol (50%/50%) with penicillin (2 × 10^6^ U/L), streptomycin (200 mg/L), and amphotericin B (250 mg/L) and kept at 4 °C during transport until reaching the lab by the end of each sampling day, then stored at −80 °C until use.

### 2.2. Virus Detection and Isolation

Viral nucleic acids were extracted using the MagMax viral/pathogen nucleic acid isolation kit (Applied Biosystems, Waltham, MA, USA) using the Kingfisher Flex Purification System as per manufacturer instructions. A total of 200 µL of each sample was extracted in a class III biosafety cabinet and then eluted in 50 µL elution buffer. Influenza A was detected for all samples using the WHO real-time RT-PCR membrane gene primers (M) assay using AgPath-ID One-Step RT-PCR kit (Applied Biosystems) [23]. Positive influenza A samples were subjected to viral isolation in 10–12-day-old SPF embryonated chicken eggs (SPF-ECE) (Koum Oshiem SPF chicken farm, Fayoum, Egypt). Influenza isolates were determined by hemagglutination activity using 0.5% chicken red blood cells [24]. Viruses were titrated by 50% egg infectious dose (EID_50_) and 50% tissue culture infectious dose (TCID50) according to the method of Reed and Muench [25].

### 2.3. Sequencing and Sequence Analysis

The first strand cDNA of H5N1 RNA isolates was generated using the first-strand synthesis Superscript IV kit (Invitrogen, Waltham, MA, USA) and the universal conserved Uni12/13 primers, then amplified with the primers for the influenza A full eight gene segments [26]. DNA amplicons were purified using GFX PCR DNA and gel bands purification kit (Cytiva Life Sciences, Marlborough, MA, USA). DNA sequence libraries were prepared using Nextera XT DNA-Seq library prep kits (Illumina, San Diego, CA, USA) according to the manufacturer’s instructions. Pooled libraries were sequenced using the MiniSeq Illumina genome sequencer with 150 nucleotide paired-end reads. Using reference genomes, sequencing reads were further analyzed using the CLC genomic workbench (Qiagen, Hilden, Germany). H5N1 viral sequences were aligned with Egyptian H5N1 sequences along with reference genomes retrieved from GISAID database using the ClustalW multiple alignment accessory application (BioEdit Sequence alignment editor version 7.2.5). Neighbor-joining phylogenetic tree for each genome segment was constructed using the MEGA X software [27], using Kimura’s two-parameter distance model and 1000 bootstraps. Accession numbers are listed in Table S1.

### 2.4. Viral Replication Kinetics in Mammalian Cell Lines

Madin-Darby canine kidney (MDCK) cells (ATCC, CCL-34) and human lung epithelial (A549) cells (ATCC, CCL-185) were obtained from the Center of Excellence for Influenza Research and Surveillance at St. Jude Children’s Research Hospital (Memphis, TN, USA) and used to determine the growth kinetics of two H5N1 isolates. A/Pintail/Egypt/RA19853OP/2021 (RA19853OP) and A/duck/Egypt/BA20361C/2022 (BA20361C) viruses were titrated in MDCK cells by plaque assay [24]. MDCK and A549 cells were grown in six-well plates with Dulbecco’s Modified Eagle’s Medium (DMEM) supplemented with 1% antibiotic-antimycotic mixture and 5% inactivated fetal bovine serum (FBS) (Gibco, Waltham, MA, USA) at 37 °C and 5% CO_2_ in a humidified incubator. Confluent 80–90% cell monolayers were infected with 0.05 multiplicity of infection (MOI). After one hour of incubation at 37 °C, the viral inoculum was removed, cells were replenished with 3 mL/well of maintenance media (DMEM supplemented with 1% antibiotic mix (Gibco) and 4% bovine serum albumin (BSA) (Sigma, St. Louis, MO, USA), four replicates of the infected cells supernatants were harvested at 12, 24, 36, and 48 h post-infection (hpi), viral growth was determined with TCID50 assay then calculated using the Reed-Muench method [25].

### 2.5. Animal Experiment

#### 2.5.1. Ethical Aspect

The National Research Center Research Ethics Committee approved all the animal studies (approval no: 06210123, approval date: 5 January 2023). All animal experiments were conducted in class III isolators. H5N1 experiments were performed under the guidelines of the Animal Care and Use Committee. Animals were anesthetized with ketamine anesthesia, and all efforts were made to minimize suffering. During the experiments, the animals would be euthanized via CO_2_ asphyxiation if they manifested severe symptoms, such as inactivity, loss of appetite, or loss of 30% or more of body weight.

#### 2.5.2. Pathogenicity, Virulence, and Viral Shedding in SPF Chickens

A total of 120 SPF four-week-old Lohmann LSL white layers, purchased from Koum Oshiem SPF chicken farm, were divided into five groups (four for infection and one uninfected control). Two groups were infected intranasally with 0.5 mL of 10^6^ EID_50_ of RA19853OP and BA20361C respectively, the other two infection groups were infected with 10^4^ EID_50_/0.5mL of the same viruses, and the control group was inoculated with 0.5 mL PBS/chicken. In each cage, 13 chickens were inoculated with the corresponding virus (donor) and seven non-infected chickens (contact) were added to each infected group at 6 hpi. Cloacal and oropharyngeal swabs were collected from survived chickens at 1, 3, and 5 days post inoculation (dpi) for viral shedding assessment. At 3 and 5 dpi, two chickens of each group were humanely euthanized, and lungs, kidneys, intestines, and brains were collected for viral shedding examination and histopathological studies. We homogenized 0.1 g of each organ in 1 mL sterile PBS using the tissue lyse LT (Qiagen), spun at 1000× *g* for 10 min. Viral RNA was extracted from the swab samples and tissue homogenates using the MagMax viral/pathogen nucleic acid isolation kit (Applied Biosystems) and the Kingfisher Flex Purification System, and tested by RT-qPCR using primers and probes specific for the M gene. A 10-fold dilution series of titrated BA20361C and/or RA19853OP (known EID_50_ titer) virus was used to calculate viral titers, which are displayed as relative equivalency units (REUs), as previously described [28]. For histopathology, the 10% neutral buffered formalin solution-fixed organs were embedded in paraffin, sliced, fixed on microscopical slides, stained with hematoxylin and eosin (H&E), and examined histologically with a four-grade histopathological scale. The histopathological lesions were scored on a 0–4 scale. In the lung sections, a score of 0 indicates no lesion; 1, mild inflammation; 2, moderate inflammatory infiltration; 3, multifocal perivascular, peribronchiolar, or alveolar inflammation; and 4, diffuse parenchymal pneumonia with consolidation and/or hemorrhage. In the intestine, a score of 0 indicates normal mucosa; 1, mild degeneration of the villi; 2, moderate desquamation of epithelial cells; 3, focal lymphoid infiltration with eroded patches; and 4, marked villous atrophy and degenerated crypts. The histological lesions in the renal tissue (including glomerular atrophy, tubular epithelial exfoliation, and lymphoid infiltration) and the cerebral cortex (including neuronal apoptosis, microgliosis, and vascular congestion/dilatation) were also scored on a 0–4 scale (0 = no lesions, 1 = mild, 2 = moderate, 3 = severe, and 4 = very severe), as previously described [29]. The histopathological scoring was performed by two independent pathologists who were blinded to the groups.

#### 2.5.3. Statistical Analysis

Significance of differences between tested groups and the control group was calculated using GraphPad Prism V8.0 software (GraphPad Inc., San Diego, CA, USA) by ANOVA with Bonferroni post hoc testing. Chi-square test was used to analyze the Epizootic variables, and Kaplan-Meyer test was used to compare survival. *p*-values < 0.05 were considered statistically significant.

## 3. Results

### 3.1. H5N1 Virus in Live Bird Markets

Overall, 20.94% (484/2311) of all tested samples were positive for influenza A. Influenza A was detected more significantly in oropharyngeal than cloacal samples, with 23.83% and 16.6%, respectively (*p* < 0.0001). Influenza A was detected in 17.26% of environmental surface samples, 31.84% of birds’ drinking water, and 18.18% of birds’ ambient air samples (Table 1). We identified 17 isolates as H5N1 with complete genome sequencing. H5N1 viruses were originally collected from different study sites from apparently healthy birds, mostly from ducks (four isolates from domestic ducks, seven from Muscovy ducks, and three from garganey ducks), one chicken, and one pigeon.

### 3.2. Phylogenetic Analysis and Sequence Similarity

**Figure 1:**
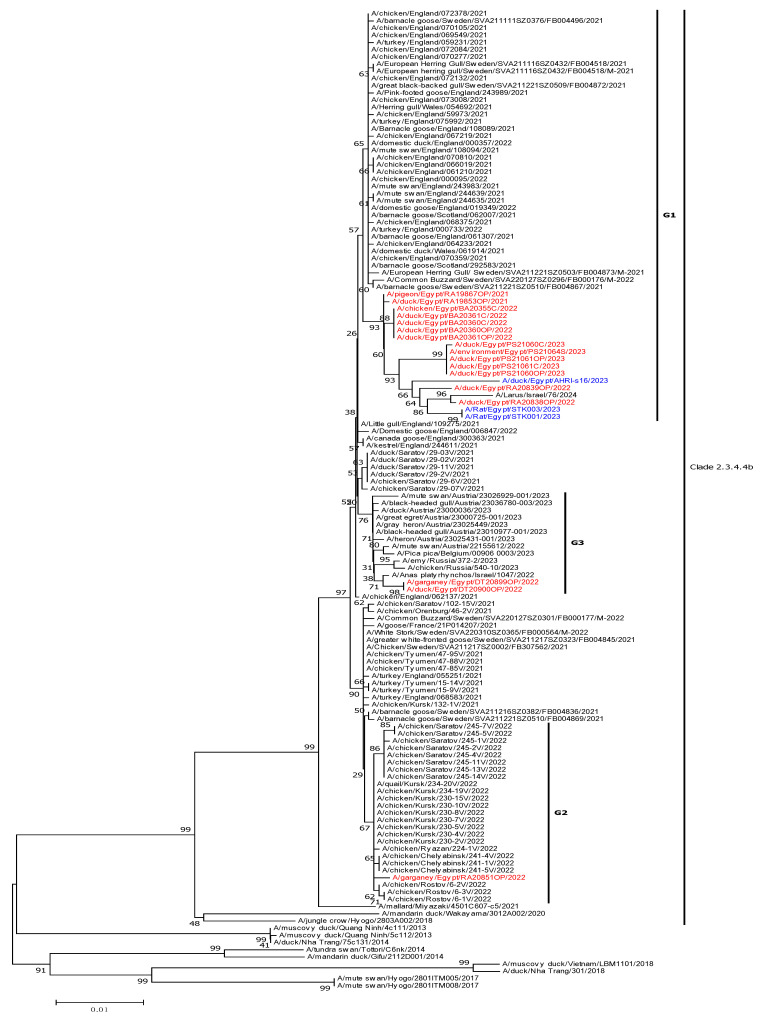

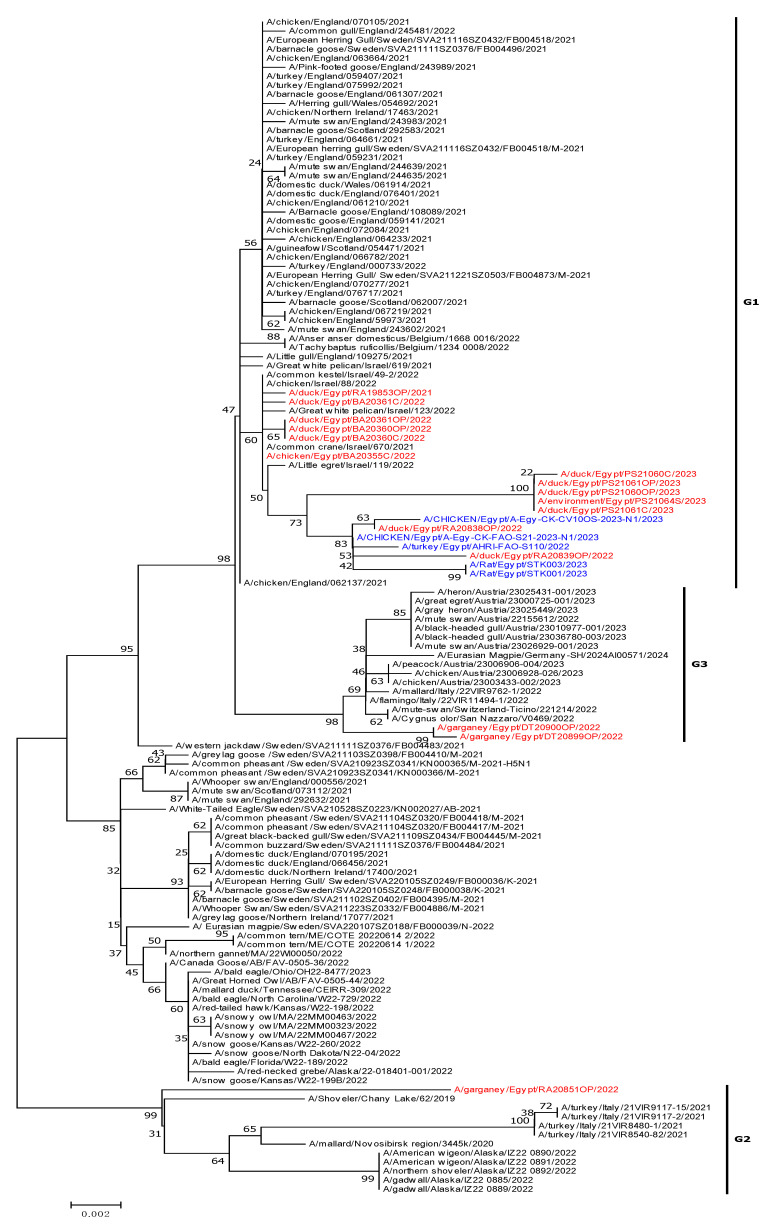
Phylogenetic analysis of HA and NA genes of HPAI H5N1viruses detected in the Egyptian LBM between November 2021 and March 2023 as compared to reference gene sequences using MEGA X software through Neighbor-joining phylogenetic tree using Kimura’s two-parameter distance model and 1000 bootstraps. The viruses isolated in this study are shown in red and other Egyptian viruses are shown in blue.

Phylogenetic analysis of the HA gene revealed that Egyptian H5N1 HPAI viruses were closely related to recent HPAI viruses isolated from Eurasian countries. All isolates belonged to clade 2.3.4.4b and formed three distinct genetic groups (G1–G3), suggesting separate introductions of three genetically distinct viruses (Figure 1).

Viruses of the G1 cluster were closely related to clade 2.3.4.4b H5N1 HPAI viruses that shared a common ancestor with viruses detected in Europe and the Middle East [15]. Those appeared in 2021 and continued circulating in poultry in different sites, and most isolates from 2022–2023 were from the same cluster (Figure 1). A G2 virus was introduced by wild birds in 2022 and was closely related to a H5N1 HPAI virus identified in Russia in 2022. Two viruses, A/garganey/Egypt/DT20899OP/2022 and A/garganey/Egypt/DT20900OP/2022, were isolated in 2022 from wild birds and clustered together to form a distinct genetic group, G3, along with other viruses detected in the Middle East and Europe.

Similarly, the NA gene was also divided into three genetic groups (G1–G3) that were closely related to HPAI viruses identified in Eurasia (Figure 1). The phylogenetic analysis of the six internal viral genes was similar to the HA and NA genes of H5N1 HPAI viruses (Figure S1).

### 3.3. Molecular Characterization

We conducted analysis of amino acid sequences responsible for pathogenicity, adaptation to mammalian hosts, transmission in mammals, and antiviral resistance to understand the characteristics of Egyptian HPAI viruses during 2021–2023. The molecular characteristics of all 17 isolated H5N1 viruses were analyzed based on the whole-genome sequences. Based on the amino acid sequences of the HA genes, all 17 isolated H5N1 viruses possessed multi-basic cleavage site PLREKRRKR↓GLF (↓ denotes cleavage site), which is characteristic of HPAIVs (Table S2). The isolates had Q226 and G228 (H3 numbering) at the receptor-binding sites of the HA proteins, which suggested the avian-like α2,3-linked sialic acid (α2-3-SA) receptor binding preference. However, mammalian-adapting mutations such as the S158N (H3 numbering) substitution were detected. The E627K and D701N mutations within the PB2 protein, which are important for adaptation of AIVs to mammals [30,31], were not observed. Mutations in the NA protein, which is associated with resistance to oseltamivir (Table S2), were also not detected. None of the mutations at positions 26, 27, 30, 31, and 34 in the M2 protein that facilitate resistance to some anti-influenza drugs (i.e., M2 ion channel blockers) were detected. However, we found that all H5N1 contained P42S and V149A substitutions in the NS1 protein, which are associated with virulence and pathogenicity in mice and chickens, respectively [32,33].

### 3.4. Growth Kinetics of H5N1 Viruses in Mammalian Cells

**Figure 2:**
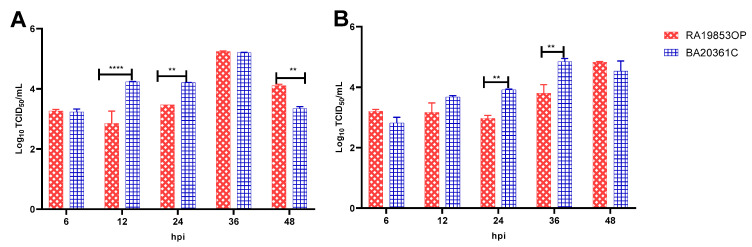
Growth kinetics of H5N1 viruses in MDCK (**A**) and A549 (**B**) cells. Cells were inoculated at a multiplicity of infection of 0.05 TCID50/cell with the A/Pintail/Egypt/RA19853OP/2021 or A/duck/Egypt/BA20361C/2022. Supernatants were collected at the indicated time points and titrated in MDCK cells by TCID50. Error bars depict standard deviations. ** *p*  <  0.01, **** *p*  <  0.0001.

To determine the replication of H5N1 viruses in mammalian cells lines, we compared the multicycle growth kinetics of the RA19853OP and BA20361C H5N1 viruses in MDCK and A549 cells. Both viruses replicated efficiently in both MDCK and A549 cells at MOI of 0.05. The viral titer of BA20361C was significantly higher than RA19853OP at 12 and 24 hpi on MDCK cells and 24 and 48 hpi on A549 cells (Figure 2). To identify the mutations potentially responsible for the increased replication of BA20361C in MDCK and A549 cells, we compared the full genomes of both viruses. Several mutations were detected in the PB1 (M688V), NP (K48R and S84N), and NA (S451V) proteins of BA20361C. Further experiments are needed to identify which of those mutations might be responsible for the differences in replication.

### 3.5. Replication and Virulence of the H5N1 Viruses in Chickens

**Figure 3:**
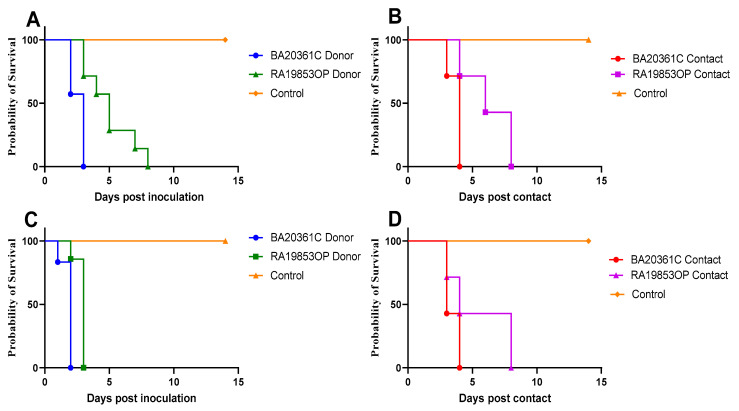
Survival curves for inoculated and contact SPF chickens with 10^4^ (**A**,**B**) and 10^6^ (**C**,**D**) EID_50_. Seven 4-week-old chickens were inoculated with the A/Pintail/Egypt/RA19853OP/2021 or A/duck/Egypt/BA20361C/2022 and seven naïve chickens were cohoused with the inoculated chickens at 6 h post inoculation. The chickens were monitored for signs of illness or death over a period of 14 d following the inoculation.

**Figure 4:**
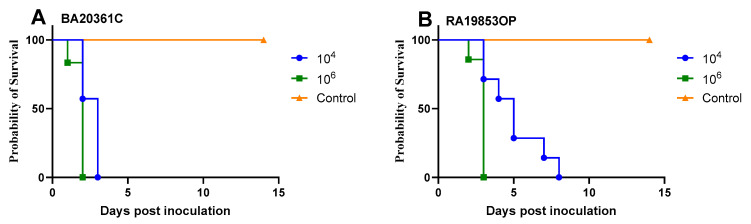
Survival curves comparing dose effect of each challenge virus in donor chickens, BA20361C (**A**) and RA19853OP (**B**). The higher dose had a significantly shorter time to death.

To investigate the pathogenicity and transmission of H5N1 viruses in SPF chickens, we inoculated groups of chickens with 10^6^ and 10^4^ EID_50_ of each virus, then contact chickens were directly added at 6 hpi to the same cage. Seven inoculated SPF chickens, as well as the seven contact chickens in either virus group, were monitored for morbidity and mortality until 14 dpi. All chickens challenged with BA20361C died at 2 dpi, while chickens challenged with RA19853OP died at 3 dpi with 10^6^ EID_50_ (Figure 3). Meanwhile, chickens challenged with 10^4^ EID_50_ of BA20361 died at 3 dpi, and RA19853OP started to die between 4 and 8 dpi (Figure 3). The higher challenge dose was faster to kill all donor chickens for both viruses with statistically significant *p*-values (Figure 4).

**Figure 5:**
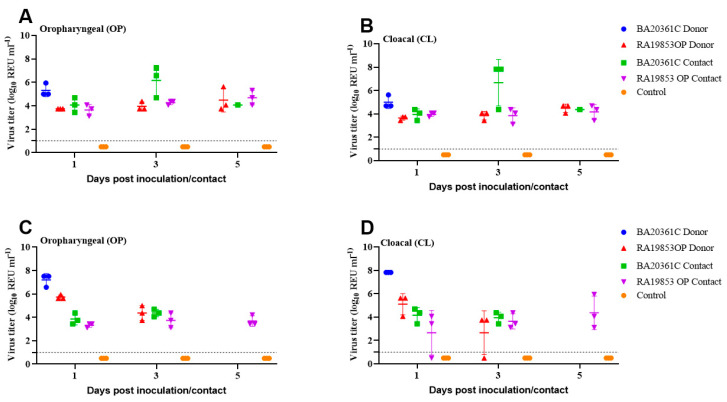
Viral shedding titers obtained from SPF chickens inoculated/contacted with 10^4^ (**A**,**B**) and 10^6^ (**C**,**D**) EID_50_ of A/Pintail/Egypt/RA19853OP/2021 or A/duck/Egypt/BA20361C/2022. Viral titers (shown as relative equivalence units [REUs] of 50% egg infectious dose [EID_50_]) were determined by using the M-gene RRT-PCR. Dotted horizontal lines indicate the M-gene RRT-PCR positive cut-off.

Virus titers in cloacal and oropharyngeal swabs collected from chickens challenged with 10^6^ EID_50_ of BA20361C rapidly peaked at 1 dpi (Figure 5). In contrast, vRNA titers in cloacal and oropharyngeal swabs of chickens challenged with 10^6^ EID_50_ of RA19853OP at 1 and 3 dpi were detected before the death of the chickens, while in the chickens challenged with 10^4^ EID_50_, the virus titers were detected until 5 dpi (Figure 5).

To investigate the transmission of H5N1 viruses, contact chickens were monitored and oropharyngeal and cloacal swabs were collected at 1, 3, and 5 dpi. The rate of transmission was 100%, and all chickens died following the exposure between 4 days post contact (dpc) and 8 dpc for both viruses, with two 10^6^ EID_50_ and 10^4^ EID_50_ (Figure 5). vRNA was detected by the oropharyngeal and cloacal swabs, and peak titer was 10^7.19^ REU of EID_50_ for the chicken challenged with 10^6^ EID_50_ of BA20361C at 1 dpi, and 10^7.82^ REU of EID_50_ for those challenged with 10^4^ EID_50_ at 3 dpc, along with the group challenged with RA19853OP (Figure 5).

**Figure 6:**
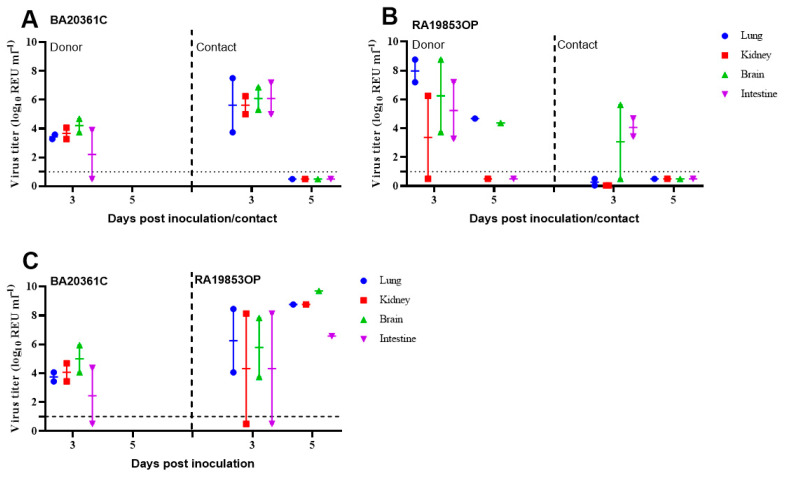
Viral titers in lung, kidney, brain, and intestine tissues of inoculated/contacted chickens at 3 and 5 days postinfection (dpi) with 10^4^ (**A**,**B**) and contacted chickens with 10^6^ (**C**) EID_50_ of A/Pintail/Egypt/RA19853OP/2021 or A/duck/Egypt/BA20361C/2022. Viral titers (shown as relative equivalence units [REUs] of 50% egg infectious dose [EID_50_]) were determined by using the M-gene RRT-PCR. Dotted horizontal lines indicate the M-gene RRT-PCR positive cut-off.

To investigate the tissue tropism of H5N1 viruses, we collected organs from inoculated and contacted chickens with 10^4^ EID_50_ of both two viruses at 3 dpi and at 5 dpi. vRNA was tested by M-gene RRT-PCR from a range of tissues obtained from two inoculated and contacted chickens. At 3 dpi, the vRNA was detected in all organs of inoculated chickens challenged with BA20361C and RA19853OP, with vRNA titers detected in lungs 10^3.27–3.59^ REU of EID_50_, kidney 10^3.27–4.06^ REU of EID_50_, brain 10^3.74–4.68^ REU of EID_50_, and intestine 10^3.9^ REU of EID_50_, while in the contact group, the virus shedding showed higher titers in all sampled organs (Figure 6A). For the RA19853OP, the vRNA was detected in all sampled organs of two chickens and detected in the kidney in one chicken with vRNA titer 10^6.25^ REU of EID_50_ (Figure 6B). At 5dpi, we show that vRNA titers were detected in the lungs and brain in chickens inoculated with RA19853OP (Figure 6B).

Tissue tropism of H5N1 viruses was investigated in SPF chickens contacted with 10^6^ EID_50_ of both viruses at 3 dpc and 5 dpc. Contacted chickens with 10^6^ EID_50_ of BA20361C and RA19853OP displayed viral replication in all internal organs collected (lungs, kidneys, brain, and intestine), with vRNA titers of 10^3.4–8.1^ REU of EID_50_ (Figure 6C). At 5 dpc, the organs were collected from one chicken of the contact group inoculated with RA19853OP, and the vRNA was detected in all organs with titers of 10^6.5–9.6^ REU of EID_50_ (Figure 6C).

### 3.6. Histopathological Assessment of H5N1 Infection in Different Chicken Organs

**Figure 7:**
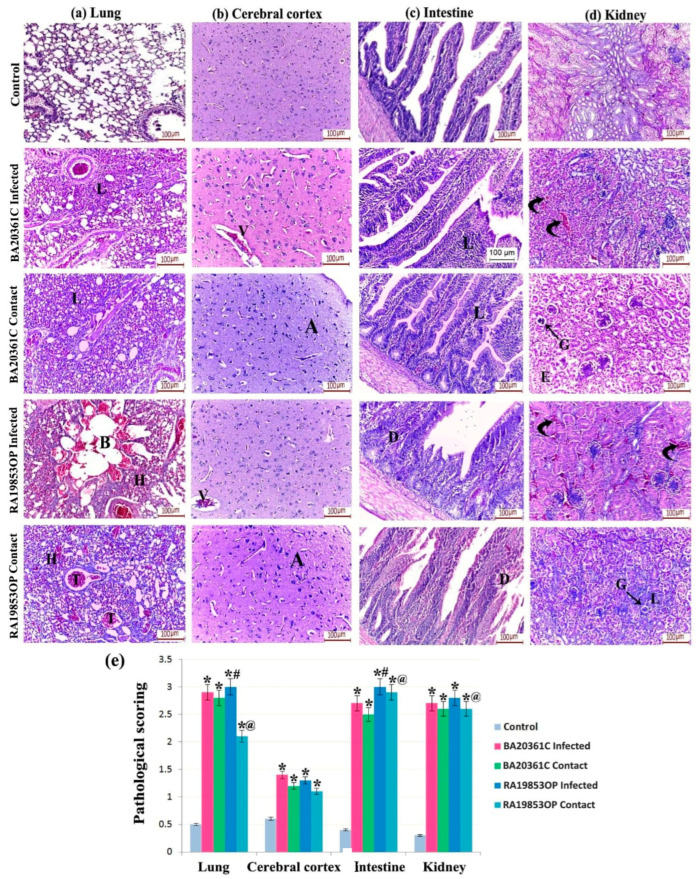
Hematoxylin and eosin (H&E) staining and pathological quantification of chicken tissue of (**a**) control group showing normal lung alveoli and bronchioles. The BA20361C infected and contact groups show marked interstitial pneumonia with lymphoid cellular infiltration (I). The RA19853OP chicken shows hemorrhagic pneumonia (H) with areas of bronchiectasis (B) in the infected group and vascular thrombosis (T) in the contact group. (**b**) The control chicken displays an intact structure of the cerebral cortex. The BA20361C and RA19853OP infected groups show vascular dilatation and congestion (V). The contact groups show numerous apoptotic neurons (A) and glial cells (G). (**c**) The intestinal tissue of the BA20361C infected and contact groups revealed lymphoid infiltration of the lamina propria (L). The RA19853OP infected and contacted chickens show degenerated crypts and villi (D). (**d**) The kidney sections of the BA20361C and RA19853OP infected chicken show marked congestion of the interstitial capillaries (curved arrows). The BA20361C and RA19853OP contacted chicken show atrophic glomeruli (G) with tubular exfoliation (E) in the BA20361C contact group and numerous lymphoid inflammatory cells (L) in the RA19853OP contact group. (**e**) Histopathological scoring. *: significant versus control, #: significant versus BA20361C infected, @: significant versus BA20361C contact at (*p* < 0.05) using ANOVA, Bonferroni post hoc testing.

Collected organs from euthanized non-infected chickens at 3 dpi were fixed, paraffin processed, and hematoxylin-eosin stained. Lungs of the uninfected controls appeared normal, with intact architecture, exhibiting normal morphological alveoli and bronchioles. The lungs of BA20361C infected and contact groups developed diffuse parenchymal pneumonia with lymphoid cellular infiltration. The RA19853OP infected chickens showed inflammatory consolidation with hemorrhagic pneumonia. There was notable bronchiectasis in the infected group and vascular thrombosis in the contact lungs (Figure 7a). The cerebral cortex of the uninfected chickens appeared structurally intact, with normal neuropil, neurons, and glial cells. The cerebral cortex of BA20361C and RA19853OP infected chickens displayed vascular dilatation and congestion. The contact groups showed numerous apoptotic neurons and glial cells (Figure 7b).

The intestinal tissue examination of the BA30361C infected and contacted chickens exhibits a lymphoid infiltration of the lamina propria, while those infected with RA19853OP and contacted chickens’ intestines appeared with degenerated crypts and villi. The control uninfected chickens’ intestines appeared to have no histopathological changes (Figure 7c). The kidneys of the BA20361C and RA19853OP infected chicken appeared with plain congestion of the interstitial capillaries. The BA20361C and RA19853OP contacted chicken unveiled atrophic glomeruli with tubular exfoliation in the BA20361C contact group and numerous lymphoid inflammatory cells in the RA19853OP contact group (Figure 7d). Overall, histopathological scores are comparable between infected and contacted chickens in both virus groups (Figure 7e).

## 4. Discussion

Egypt lies at the crossroads of the Black Sea–Mediterranean and East African–West Asian migratory flyways, which converge over the Nile Delta, bringing a high diversity of IAVs to Egypt. This convergence creates a “melting pot” of viruses, potentially leading to possible reassortment and emergence of new strains, including those with zoonotic potential. Many influenza viruses are endemic in Egyptian domestic poultry, including HPAI H5N1, H5N8, and LPAI H9N2 viruses, which, coupled with human exposure, increase the risk of reassortments and the emergence of new influenza strains.

In this study, we aimed to genetically and antigenically characterize H5N1 viruses isolated in Egypt though our active surveillance between 2021–2023. LBMs in coastal regions of Egypt are considered hotspots for virus transmission, mutation, and potential reassortment due to the direct contact between domestic poultry, captured migratory birds, stray animals, and humans. Swab samples were collected from birds and from environmental sources including birds’ drinking water, market working surfaces, animal cages, and weighing scales.

Influenza A viruses were detected in more than 20% of all samples. The majority of the H5N1 isolates were from apparently healthy ducks (one Pintail, three Garganey, four Pekin ducks, and six Muscovy ducks) highlighting the potential role of ducks in the transmission of HPAI. Besides isolation of H5 from different hosts, including migratory birds and various poultry species, phylogenetic and genetic analyses revealed that all H5N1 viruses belonged to clade 2.3.4.4b Eurasian-like viruses, forming three different genetic groups, and suggesting multiple sequential introductions of genetically distinct viruses into Egypt. The spread of these variants in multiple species and geographical locations simultaneously may facilitate reassortment events as previously reported [16,34,35].

The collective genetic characteristics of those three distinct H5N1 genetic groups with the typical cleavage site PLREKRRKR↓GLF presented the Q226 and the G228 amino acid residues at the receptor binding site of the HA that favor binding to α2,3-linked sialic acid (α2-3-SA) receptors [36,37,38]. However, some substitutions associated with mammalian adaptation were also detected, such as S158N in the HA protein [39,40,41,42] and P42S in the NS1 protein linked to enhanced virulence and pathogenicity in mammals. The L222 and S224 in HA, associated with human-like receptor binding, and E627K and D701N in PB2, associated with mammalian adaptation and increased polymerase activity, were not found in our H5N1 isolates. The characterized H5N1 viruses showed high genetic similarity to an H5N1 strain isolated from a rat in Egypt in 2023 [43], further supporting the hypothesis that these viruses are undergoing adaptation to mammalian hosts.

Antiviral resistance markers were not detected in the H5N1 isolates, including E119V, H275Y, R293K, and N295S, which are known to reduce susceptibility to neuraminidase inhibitors, predominantly oseltamivir. Resistance markers to adamantane-based antivirals like amantadine and rimantadine, including L26F, V27A, A30T, G34E, and L38F of the M protein, were absent. However, the S31N substitution, which is known to confer resistance by disrupting M2 ion channel binding to adamantane drugs was present.

Wild birds and domestic ducks are key players in the maintenance, transmission, and reassortment of influenza viruses at the wild–domestic interface [16,44,45]. We investigated viral the shedding dynamics and transmission of two isolates from wild birds and domestic ducks from a distinct genetic group, G1. The two viruses exhibited high pathogenicity and virulence in the chicken model, resulting in 100% mortality by 2 and 3 dpi with an infectious dose of 10^6^ EID_50_. Similarly, contacted chickens exposed to infected birds also experienced complete mortality, albeit with delayed onset at 4 and 8 dpi. The same trend was observed with the lower infection dose (10^4^ EID_50_), where infected chickens showed delayed mortality by one additional day. These results slightly support the dose-dependent effect on infectivity, pathology, and virulence, but not on overall survival [46,47].

Higher viral shedding was detected for BA20361C than RA19853OP in the infected chickens’ oral and cloacal swabs, indicating a high transmission rate that aligns with the observed survival rates of infected and contacted chickens. Both viruses replicated systemically in SPF chickens; the RA19853OP replicated more efficiently than the BA20361C in the organs of contact chickens infected with 10^6^ EID_50_ at 3 and 5 dpi. This trend was reversed in contact chickens at 3 dpi, where BA20361C actively replicated more than RA19853OP. Histopathological analysis showed comparable tissue damage and pathological scores between chickens infected with either virus, with minor differences observed.

In conclusion, three genetically distinct groups of HPAI H5N1 viruses were detected in poultry and migrant birds, with the competence to cause systemic infection and direct infections in chickens. Therefore, intensified virological surveillance, particularly in high-risk settings such as LBMs, where animals are in direct contact with humans, is essential, along with monitoring of the genetic and pathogenic characteristics of AIV.

## Figures and Tables

**Table 1:** Epizootic variables of H5N1 isolated viruses in live bird markets. *p*-values indicate differences in influenza A positivity between categories.

Variables	Total Tested N (%)	Positive Influenza A Realtime RT-PCR N (%)	H5N1 Isolates N (%)	*p* Value
**Sample type (Total)**	**2311 (100.00)**	**484 (20.94)**	**17 (0.736)**	<0.0001
Oropharyngeal	940 (40.67)	224 (23.83)	11 (1.17)	
Cloacal	940 (40.67)	156(16.6)	5 (0.532)	
Surface	197 (8.52)	34 (17.26)	1 (0.51)	
Water	201 (8.69)	64 (31.84)	0 (0.00)	
Air	33 (1.42)	6 (18.18)	0 (0.00)	
**Site (Total)**	**2311 (100.00)**	**484 (20.94)**	**17 (0.736)**	0.0088
Port Said	447 (19.34)	89 (19.91)	5 (1.12)	
Damietta	878 (37.99)	166 (18.9)	2 (0.91)	
Kafr El-Shaikh	461 (19.94)	130 (28.2)	5 (1.085)	
Bahira	525 (22.72)	99 (18.85)	5 (0.952)	
**Health**	**1880 (100.00)**	**380 (20.21)**	**16 (0.85)**	NS
Healthy	1878 (99.98)	379 (20.18)	16 (0.85)	
Sick	0 (0.00)	0 (0.00)	0 (0.00)	
Dead	2 (0.12)	1 (50.00)	0 (0.00)	
**Species**	**1880 (100.00)**	**380 (20.21)**	**16 (0.85)**	<0.0001
Black-legged kittiwake	6 (0.32)	2 (33.33)	0 (0.00)	
Cattle egret	2 (0.12)	0 (0.00)	0 (0.00)	
Chicken	688 (36.59)	162 (23.54)	1 (0.15)	
Common Myna	2 (0.12)	0 (0.00)	0 (0.00)	
Common pochard	62 (3.29)	7 (11.29)	0 (0.00)	
Common Quail	140 (7.44)	28 (20)	0 (0.00)	
Coot	30 (1.59)	1 (3.33)	0 (0.00)	
Duck	104 (5.53)	36 (34.61)	4 (3.99)	
Egyptian turtle dove	10 (0.53)	0 (0.00)	0 (0.00)	
Eurasian golden oriole	14 (0.74)	1 (7.14)	0 (0.00)	
Garganey	178 (9.46)	37 (20.78)	3 (1.69)	
Hoopoe	2 (0.12)	0 (0.00)	0 (0.00)	
Mallard	44 (2.34)	6 (13.63)	0 (0.00)	
Moorhen	134 (7.12)	15 (11.19)	0 (0.00)	
Muscovy Duck	10 (0.53)	8 (80)	6 (6.00)	
Northern shoveler	156 (8.29)	35 (22.43)	0 (0.00)	
Pigeon	164 (8.72)	7 (4.26)	1 (0.61)	
Pintail	86 (4.57)	21 (24.41)	1 (1.16)	
Purple swamphen	10 (0.53)	7 (70)	0 (0.00)	
Ruff	16 (0.85)	1 (6.25)	0 (0.00)	
Saker falcon	10 (0.53)	2 (20)	0 (0.00)	
Turkey	10 (0.53)	4 (40)	0 (0.00)	
Wigeon	2 (0.12)	0 (0.00)	0 (0.00)	

NS = not significant.

## Data Availability

The data supporting the findings of this study are available in the manuscript and supplementary material.

## Supplementary Materials

The following supporting information can be downloaded at: https://www.mdpi.com/article/10.3390/v17101370/s1, Table S1: GenBank Accession Number of HPAI A(H5N1) viruses sequenced in this study. Table S2: Molecular characteristics of H5N1 HPAI viruses isolated in this study. Figure S1: Phylogenetic analysis of PB2, PB1, PA, NP, M, and NS genes of HPAI H5N1 viruses detected in the Egyptian LBM between November 2021 and March 2023 as compared to reference gene sequences using MEGA X software through Neighbor-joining phylogenetic tree using Kimura’s two-parameter distance model and 1000 bootstraps.

## Abbreviations

The following abbreviations are used in this manuscript:

AIV	Avian influenza viruses
BSA	Bovine serum albumin
DPC	Days post contact
DPI	Days post inoculation
DMEM	Dulbecco’s Modified Eagle’s Medium
H&E	hematoxylin and eosin
HPAI	highly pathogenic avian influenza
LBM	live bird market
LPAI	low pathogenic avian influenza
MDCK	Madin-Darby canine kidney
SPF	specific pathogen-free
EID_50_	50% egg infectious dose
TCID_50_	50% tissue culture infectious dose
