# Supplementary material for: Genetic Characterization and Pathogenesis of Highly Pathogenic Avian Influenza Virus A (H5N1) Isolated in Egypt During 2021–2023
Source: Viruses. 2025 Oct 13;17(10):1370. doi: 10.3390/v17101370 (PMC12568289; doi:10.3390/v17101370)
Supplement: Supplementary file 1 [file viruses-17-01370-s001.zip › viruses-3898873-supplementary/Table S1.pdf]

Table S1. GenBank Accession Number of HPAI A(H5N1) viruses sequenced in this study

	**Gene ***							
Label	PB2	PB1	PA	HA	NP	NA	MP	NS
A/Pintail/Egypt/RA19853OP/2021 (H5N1)	OP590530	OP590531	OP590532	OP590533	OP590534	OP590535	OP590536	OP590537
A/Pigeon/Egypt/RA19867OP/2021 (H5N1)				OR783378			OR783379	OR783380
A/Chicken/Egypt/BA20355C/2022 (H5N1)					OR783370		OR783371	OR783372
A/Duck/Egypt/BA20360C/2022 (H5N1)	OP590394	OP590395	OP590396	OP590397	OP590398	OP590399	OP590400	OP590401
A/Duck/Egypt/BA20360OP/2022 (H5N1)	OP590410	OP590411	OP590412	OP590413	OP590414	OP590415	OP590416	OP590417
A/Duck/Egypt/BA20361OP/2022 (H5N1)	OP590514	OP590515	OP590516	OP590517	OP590518	OP590519	OP590520	OP590521
A/Duck/Egypt/BA20361C/2022 (H5N1)					OR783367		OR783368	OR783369
A/Muscovy Duck/EgyptRA20838OP/2022(H5N1)	OR783411	OR783412	OR783413	OR783414	OR783415	OR783416	OR783417	OR783418
A/Muscovy Duck/EgyptRA20839OP/2022(H5N1)	OR783441	OR783442	OR783443	OR783444	OR783445	OR783446	OR783447	OR783448
A/Garganey/EgyptRA20851OP/2022(H5N1)	OR783381	OR783382	OR783383	OR783384	OR783385	OR783386	OR783387	OR783388
A/Garganey/EgyptDT20899OP/2022(H5N1)	OR783397	OR783398	OR783399	OR783400	OR783401	OR783402	OR783403	OR793319
A/Garganey/EgyptDT20900OP/2022(H5N1)	OR783341						OR783342	OR783343
A/Muscovy Duck/EgyptPS21060OP/2023(H5N1)	OR783333	OR783334	OR783335	OR783336	OR783337	OR783338	OR783339	OR783340
A/Muscovy Duck/EgyptPS21060C/2023(H5N1)	OR783433	OR783434	OR783435	OR783436	OR783437	OR783438	OR783439	OR783440
A/Muscovy Duck/EgyptPS21061OP/2023(H5N1)	OR783326	OR783327	OR783328	OR783329	OR783330	OR797352	OR783331	OR783332
A/Muscovy Duck/EgyptPS21061C/2023(H5N1)	OR783404	OR783405	OR793320	OR783406	OR783407	OR783408	OR783409	OR783410
A/Environment/EgyptPS21064S/2023(H5N1)	OR783449	OR783450	OR783451	OR783452	OR783453	OR783454	OR783455	OR783456

* PB2, basic polymerase 2; PB1, basic polymerase 1; PA, acidic polymerase; HA, hemagglutinin; NP, nucleoprotein; NA, neuraminidase; MP, matrix protein; NS, nonstructural protein.
