# Supplementary material for: Genetic Characterization and Pathogenesis of Highly Pathogenic Avian Influenza Virus A (H5N1) Isolated in Egypt During 2021–2023
Source: Viruses. 2025 Oct 13;17(10):1370. doi: 10.3390/v17101370 (PMC12568289; doi:10.3390/v17101370)
Supplement: Supplementary file 1 [file viruses-17-01370-s001.zip › viruses-3898873-supplementary/Table S2.pdf]

Table S2. Molecular characteristics of H5N1 HPAI viruses isolated in this study

Viruses	HA					NA				PB1-F1	PA		
	Cleavage site	S158N	T160A	Q226L	G228S	E119V	H275Y	R293K	N295S	N66S	V100A	S409N	A515T
A/duck/Egypt/RA19853OP/2021	PLREKRRKR/GLF	N	T	Q	G	E	H	R	N	S	V	S	T
A/pigeon/Egypt/RA19867OP/2021	PLREKRRKR/GLF	N	T	Q	G	E	H	R	N	S	V	S	T
A/duck/Egypt/BA20361OP/2022	PLREKRRKR/GLF	N	T	Q	G	E	H	R	N	S	V	S	T
A/duck/Egypt/BA20360OP/2022	PLREKRRKR/GLF	N	T	Q	G	E	H	R	N	S	V	S	T
A/duck/Egypt/BA20360C/2022	PLREKRRKR/GLF	N	T	Q	G	E	H	R	N	S	V	S	T
A/garganey/Egypt/DT20899OP/2022	PLREKRRKR/GLF	N	T	Q	G	E	H	R	N	S	V	S	T
A/garganey/Egypt/RA20851OP/2022	PLREKRRKR/GLF	N	T	Q	G	E	H	R	N	S	V	S	T
A/duck/Egypt/RA20838OP/2022	PLREKRRKR/GLF	N	T	Q	G	E	H	R	N	S	V	S	T
A/duck/Egypt/RA20839OP/2022	PLREKRRKR/GLF	N	T	Q	G	E	H	R	N	S	V	S	T
A/duck/Egypt/DT20900OP/2022	PLREKRRKR/GLF	N	T	Q	G	E	H	R	N	S	V	S	T
A/chicken/Egypt/BA20355C/2022	PLREKRRKR/GLF	N	T	Q	G	E	H	R	N	S	V	S	T
A/duck/Egypt/BA20361C/2022	PLREKRRKR/GLF	N	T	Q	G	E	H	R	N	S	V	S	T
A/environment/Egypt/PS21064S/2023	PLREKRRKR/GLF	N	T	Q	G	E	H	R	N	S	V	S	T
A/duck/Egypt/PS21060C/2023	PLREKRRKR/GLF	N	T	Q	G	E	H	R	N	S	V	S	T
A/duck/Egypt/PS21060OP/2023	PLREKRRKR/GLF	N	T	Q	G	E	H	R	N	S	V	S	T
A/duck/Egypt/PS21061C/2023	PLREKRRKR/GLF	N	T	Q	G	E	H	R	N	S	V	S	T
A/duck/Egypt/PS21061OP/2023	PLREKRRKR/GLF	N	T	Q	G	E	H	R	N	S	V	S	T

	PB2							M					NS			
	L89V	G309D	T339K	I504V	A588V	E627K	D701N	L26F	V27A	A30T	S31N	G34E	L38F	P42S	D92E	V149A
A/duck/Egypt/RA19853OP/2021	V	D	K	V	A	E	D	L	V	A	N	G	L	S	E	A
A/pigeon/Egypt/RA19867OP/2021	V	D	K	V	A	E	D	L	V	A	N	G	L	S	E	A
A/duck/Egypt/BA20361OP/2022	V	D	K	V	A	E	D	L	V	A	N	G	L	S	E	A
A/duck/Egypt/BA20360OP/2022	V	D	K	V	A	E	D	L	V	A	N	G	L	S	E	A
A/duck/Egypt/BA20360C/2022	V	D	K	V	A	E	D	L	V	A	N	G	L	S	E	A
A/garganey/Egypt/DT20899OP/2022	V	D	K	V	A	E	D	L	V	A	N	G	L	S	E	A
A/garganey/Egypt/RA20851OP/2022	V	D	K	V	A	E	D	L	V	A	N	G	L	S	E	A
A/duck/Egypt/RA20838OP/2022	V	D	K	V	A	E	D	L	V	A	N	G	L	S	E	A
A/duck/Egypt/RA20839OP/2022	V	D	K	V	A	E	D	L	V	A	N	G	L	S	E	A
A/duck/Egypt/DT20900OP/2022	V	D	K	V	A	E	D	L	V	A	N	G	L	S	E	A
A/chicken/Egypt/BA20355C/2022	V	D	K	V	A	E	D	L	V	A	N	G	L	S	E	A
A/duck/Egypt/BA20361C/2022	V	D	K	V	A	E	D	L	V	A	N	G	L	S	E	A
A/environment/Egypt/PS21064S/2023	V	D	K	V	A	E	D	L	V	A	N	G	L	S	E	A
A/duck/Egypt/PS21060C/2023	V	D	K	V	A	E	D	L	V	A	N	G	L	S	E	A
A/duck/Egypt/PS21060OP/2023	V	D	K	V	A	E	D	L	V	A	N	G	L	S	E	A
A/duck/Egypt/PS21061C/2023	V	D	K	V	A	X	D	L	V	A	N	G	L	S	E	A

A/duck/Egypt/PS21061OP/2023	V	D	K	V	A	E	D	L	V	A	N	G	L	S	E	A
