# Supplementary figures and images for: Genetic Characterization and Pathogenesis of Highly Pathogenic Avian Influenza Virus A (H5N1) Isolated in Egypt During 2021–2023
Source: Viruses. 2025 Oct 13;17(10):1370. doi: 10.3390/v17101370 (PMC12568289; doi:10.3390/v17101370)

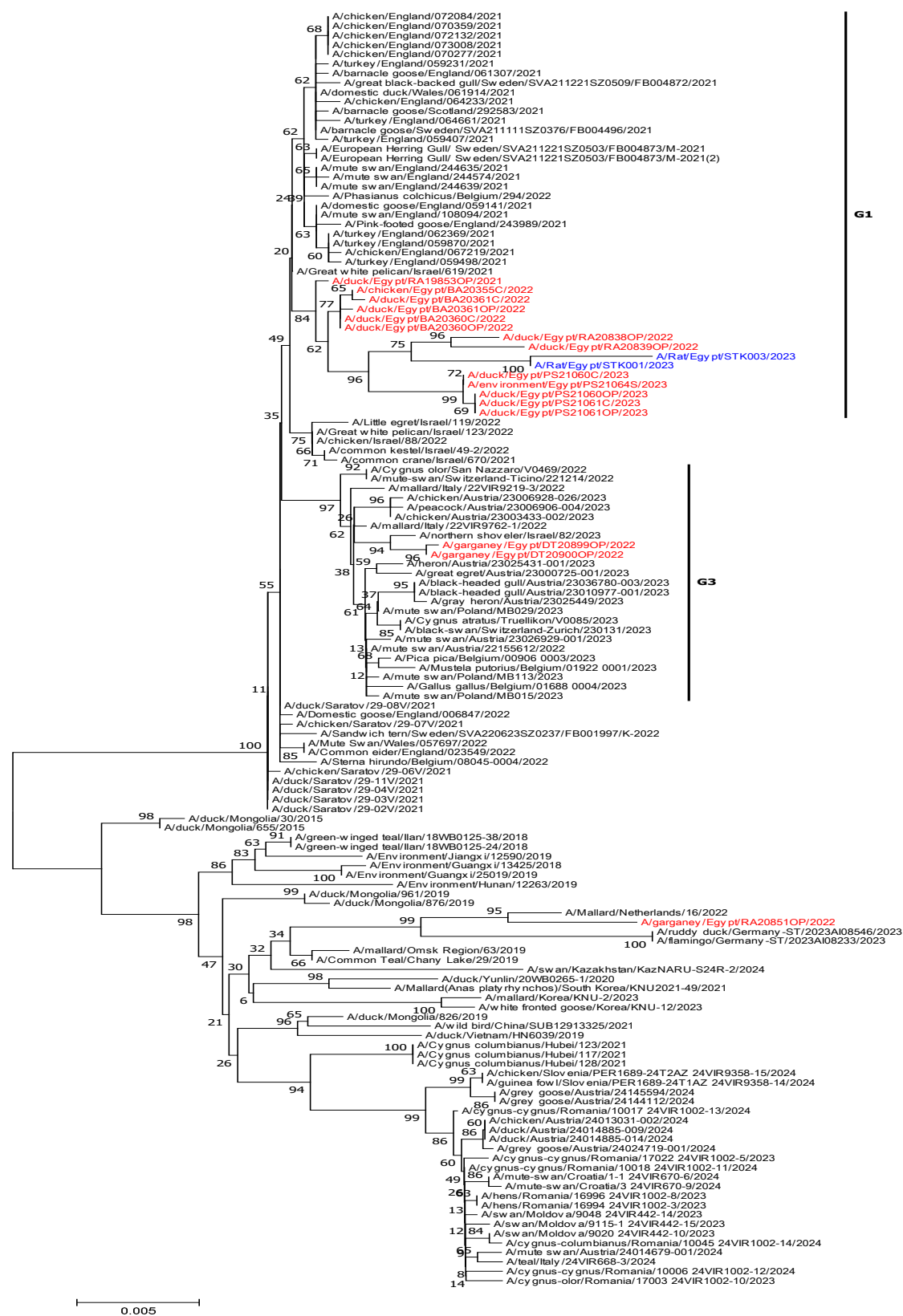

PB1

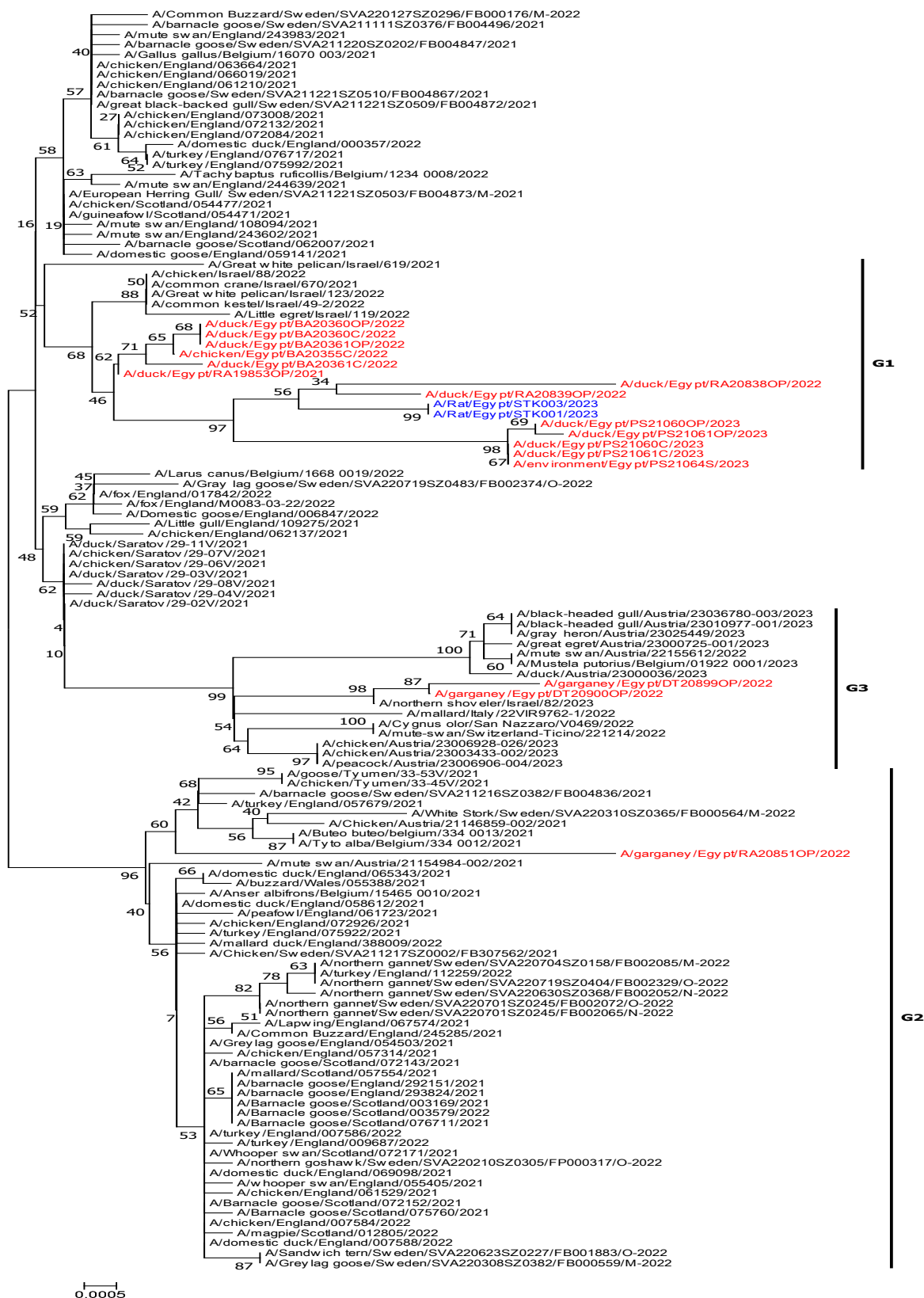

PA

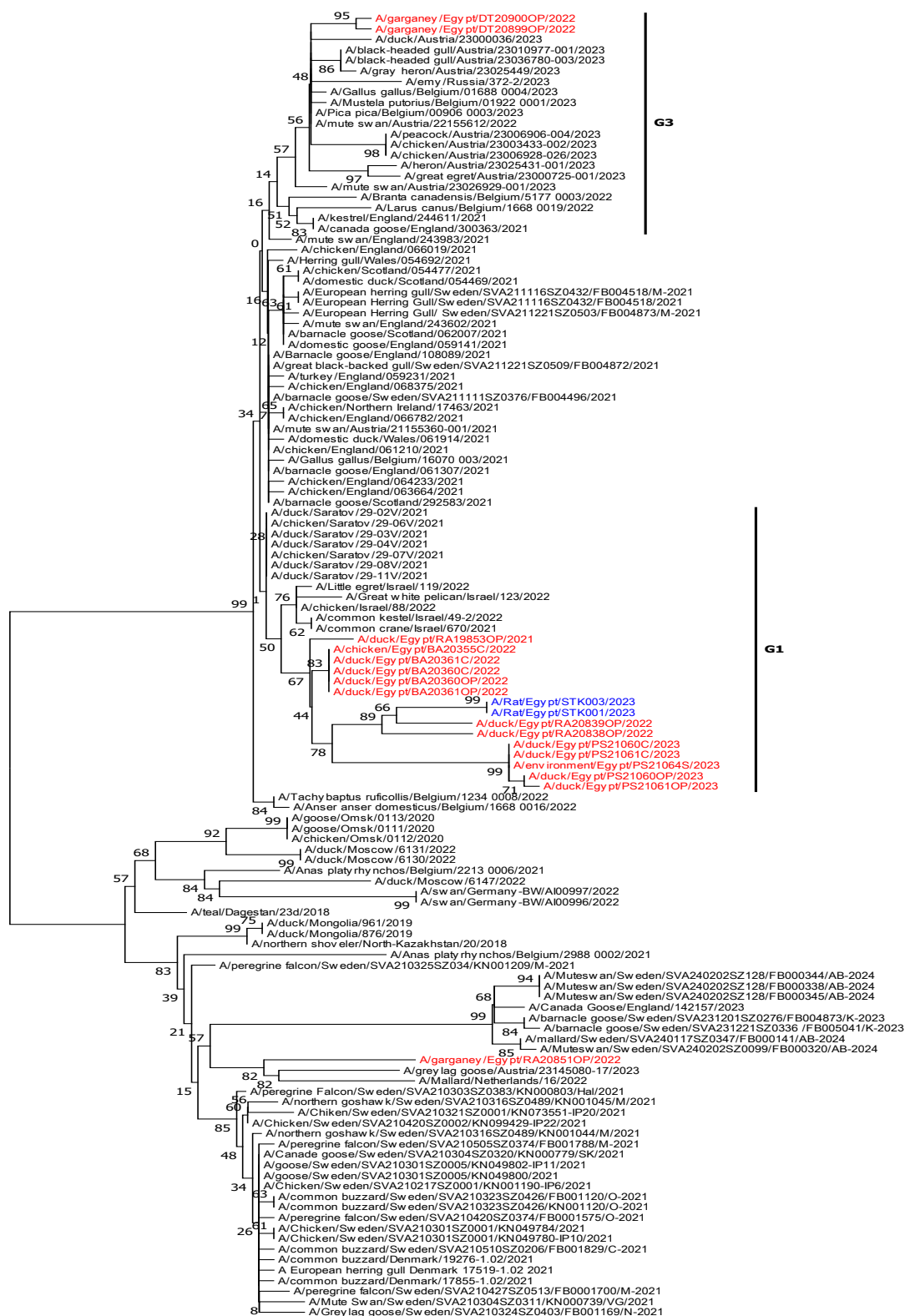

NP

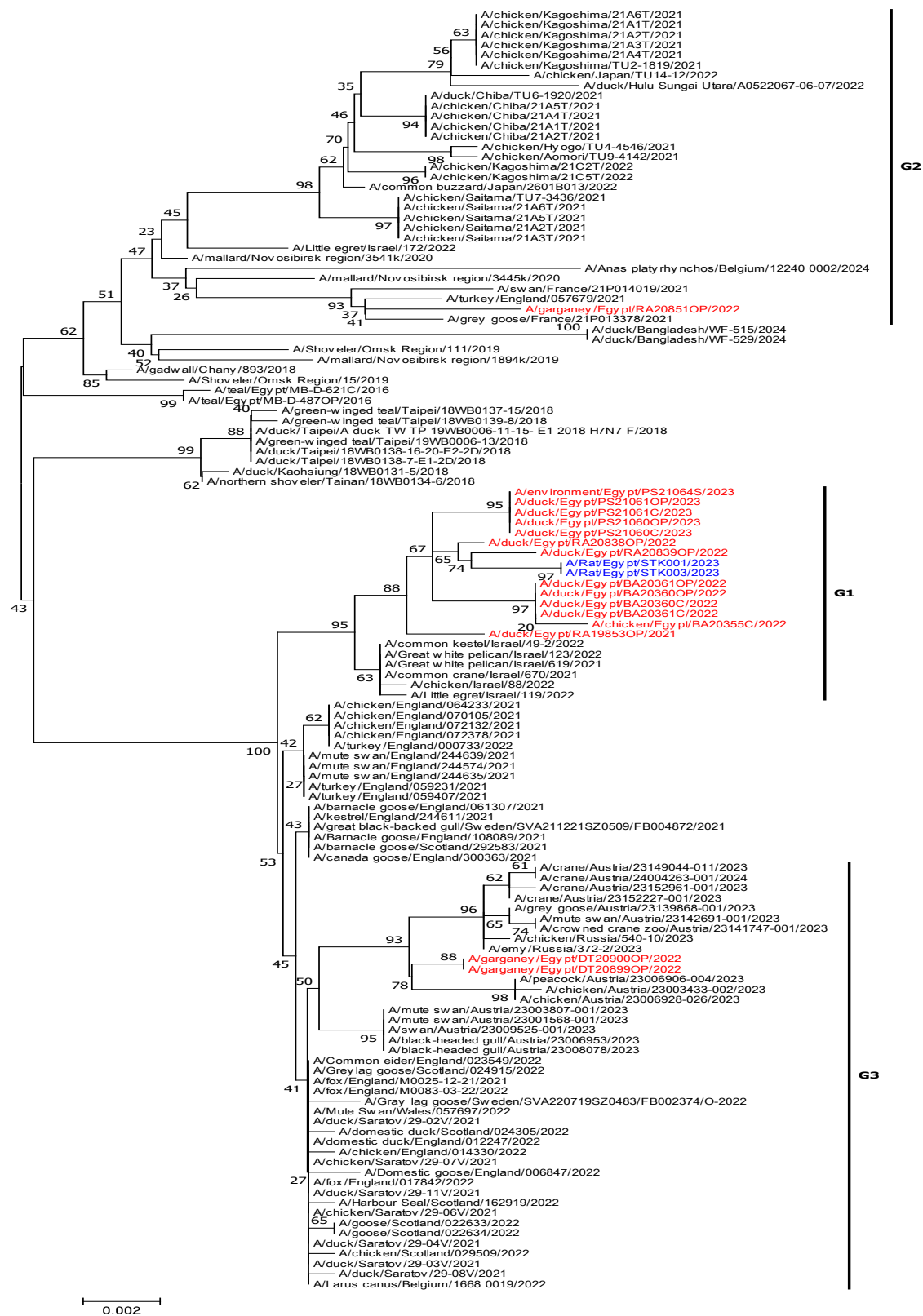

M

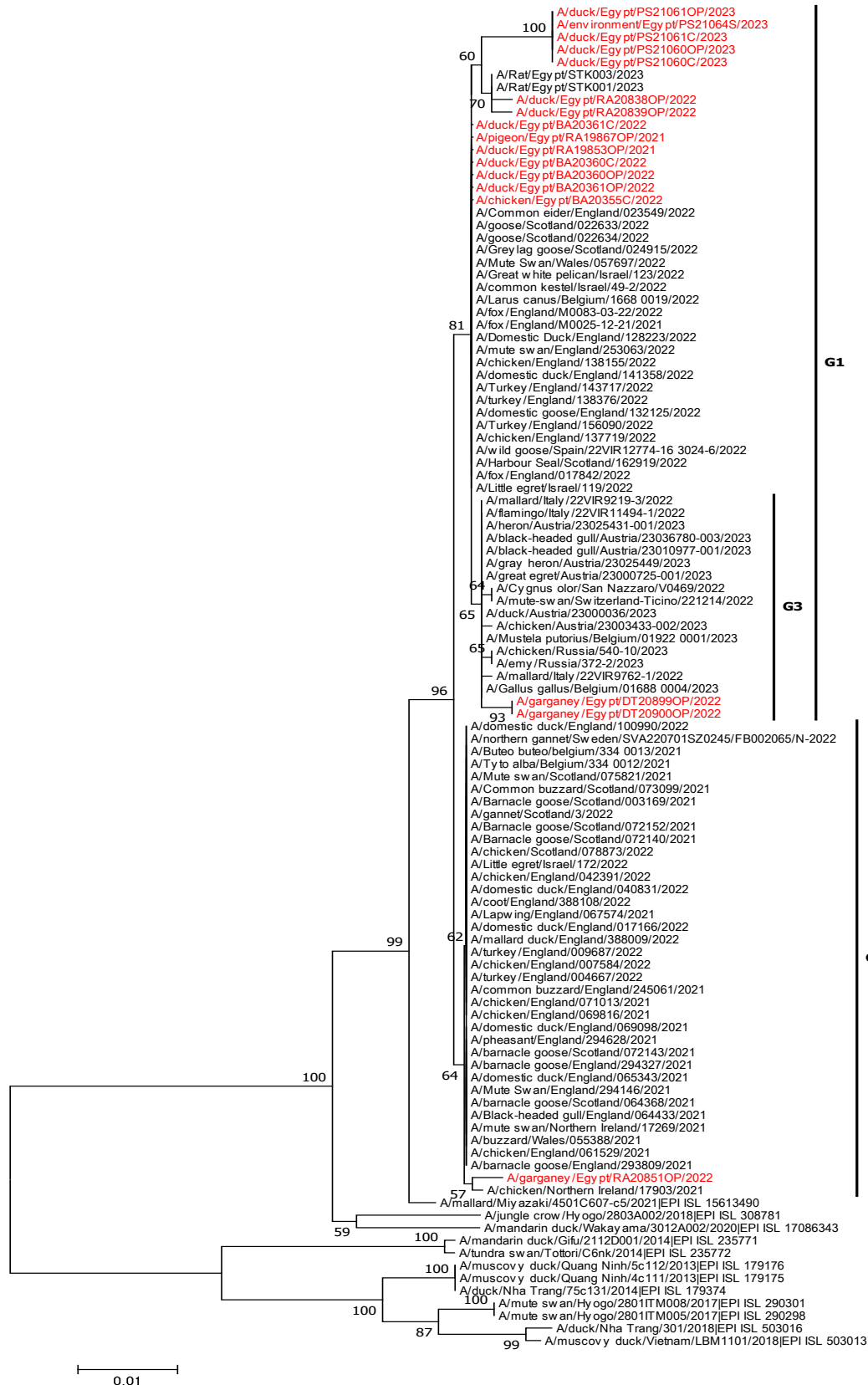

NS

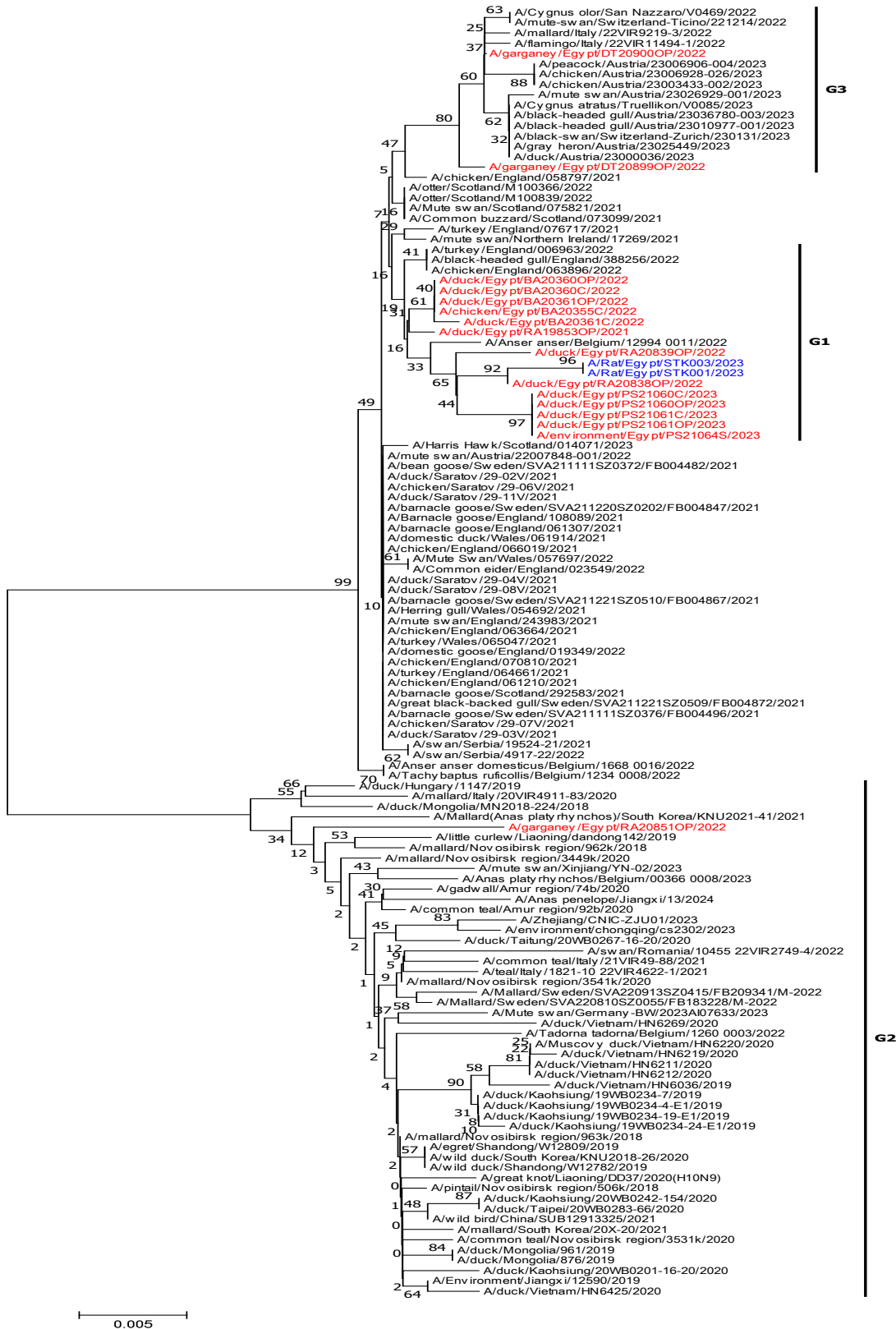

Supplement: Supplementary file 1 [file viruses-17-01370-s001.zip › viruses-3898873-supplementary/Figure S1.pdf]
